# Foreign bodies in the sigmoid colon of a psychiatric patient following self-mutilation: a case report

**DOI:** 10.1186/s13256-016-1044-y

**Published:** 2016-09-17

**Authors:** Hailu Wondimu Gebresellassie

**Affiliations:** Department of Surgery, School of Medicine, College of Health Sciences, AAU, Addis Ababa, Ethiopia

**Keywords:** Foreign bodies, Gastrointestinal, Laparotomy, Psychiatric, Patient, Self-inflicted, Case report

## Abstract

**Background:**

The act of deliberate injury to one’s own body without the help of others is a well-known phenomenon in psychiatric patients. Insertion of foreign bodies into one or more orifices is not uncommon but insertion into a body cavity or the gastrointestinal tract by self-inflicted injury is quite rare.

**Case presentation:**

A 32-year-old Ethiopian psychiatric patient presented with left lower abdominal pain of three months’ duration following the insertion of foreign bodies via a self-inflicted wound in the left lower quadrant of his abdomen. Radiological evaluation demonstrated the presence of foreign bodies. A laparotomy revealed two metallic and three wooden materials in his sigmoid colon and a hole in his sigmoid that was tightly sealed with omentum. The foreign bodies were successfully removed, the hole was closed primarily, and our patient was discharged uneventfully.

**Conclusions:**

This case illustrates that a foreign body can be inserted into the colon through a self-inflicted wound in psychiatric patients, and patients may present months later without having developed generalized peritonitis.

## Background

Foreign bodies have been reported in various parts and levels of the gastrointestinal tract. Most commonly they are swallowed accidentally or intentionally, or introduced into the rectum for various purposes [1].

Insertion of foreign bodies as a deliberate act or form of self-injurious behavior is known to occur in psychiatric patients; these foreign bodies are often inserted into one or more orifices and are usually not fatal [2].

In this report, foreign bodies are described in the sigmoid colon of a 32-year-old psychiatric patient who inserted three wooden and two metallic materials through a self-inflicted wound in his left lower abdomen in a suicide attempt.

## Case presentation

I report the case of a 32-year-old Ethiopian male patient from Addis Ababa who presented with lower abdominal pain of three months’ duration.

The abdominal pain started after our patient inserted metallic and wooden materials via a self-inflicted stab wound from a rusted knife into his left lower abdomen, 3 months prior to his presentation to our hospital. He stated that he did this to commit suicide. He did not seek any medical attention at that time because he had no family around and lived on the street. He claimed that he did not take any medication for the pain or the wound.

Our patient was taken to a clinic run by Missionaries of Charity in Addis Ababa by police after they saw the wound. He was then referred to our hospital for better management. Three months earlier, he had visited a psychiatric hospital for his psychiatric problems. He had not received any treatment because he had been asked to bring a relative with him to make sure he took his medications properly. He claimed that he occasionally used Khat (Khat is the fresh leaves and twigs of the shrub *Catha edulis*, which has a stimulating and euphoric effect when chewed or brewed as tea). He was single and had completed 12th grade in school.

**Fig. 1:**
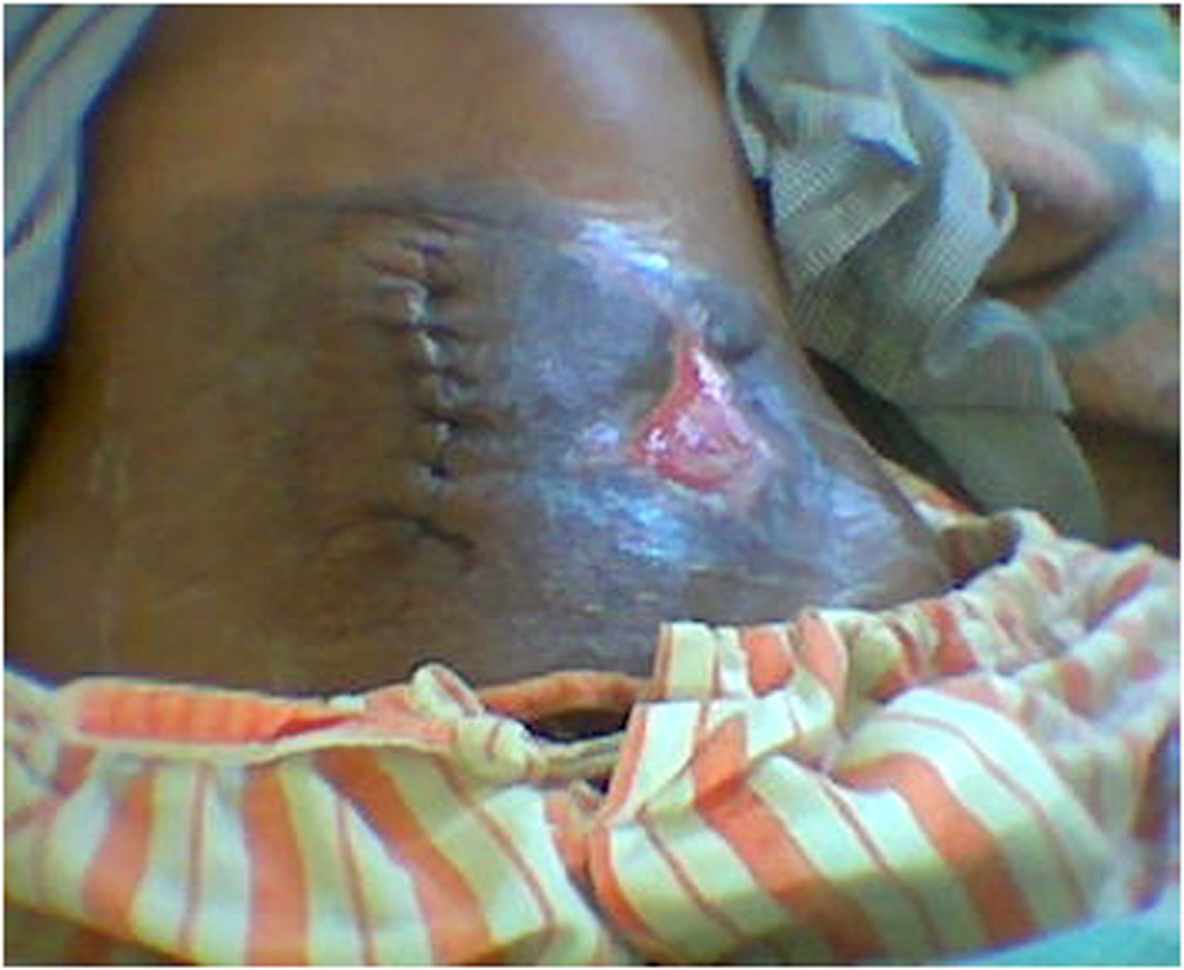
Self-inflicted wound and midline laparotomy wound. Shows a healing wound with granulating tissue in the left lower abdomen with a skin defect and a lower abdominal midline surgical wound

On examination, he was not sick looking, was indifferent to his surroundings, and had stable vital signs. The pertinent finding was of a clean, healing granulating wound in his left lower abdomen measuring 2×2 cm with skin loss. There was tenderness on deep palpation in his lower abdomen but no rebound tenderness (Fig. 1).

**Fig. 2:**
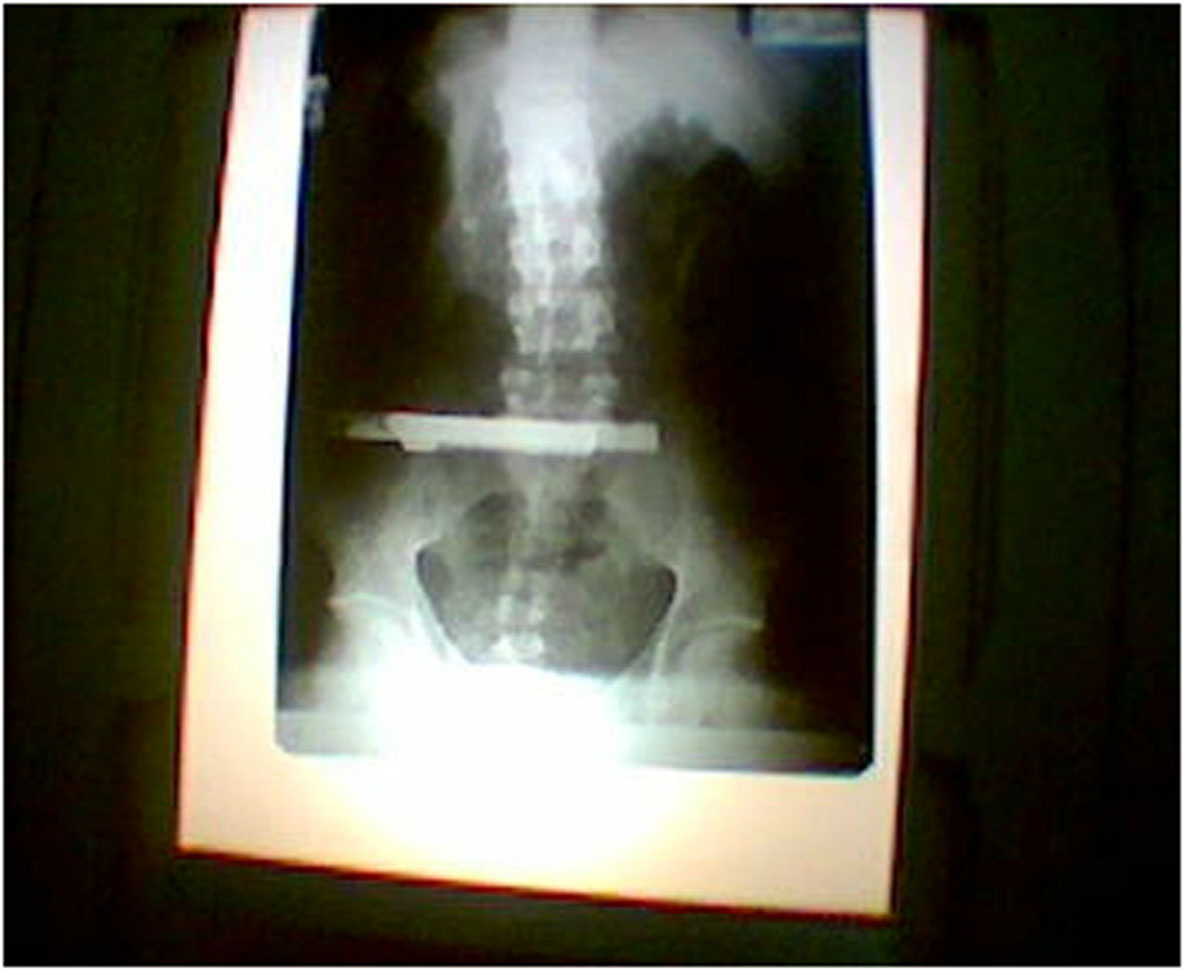
Plain film of abdomen. X-ray shows radio-opaque materials lying transversely in the lower abdomen

Radiographic investigations revealed two radio-opaque shadows on the plain film of his abdomen, estimated to be 12–15 cm long (Fig. 2). We diagnosed intra-abdominal foreign bodies and opted for exploratory surgery.

**Fig. 3:**
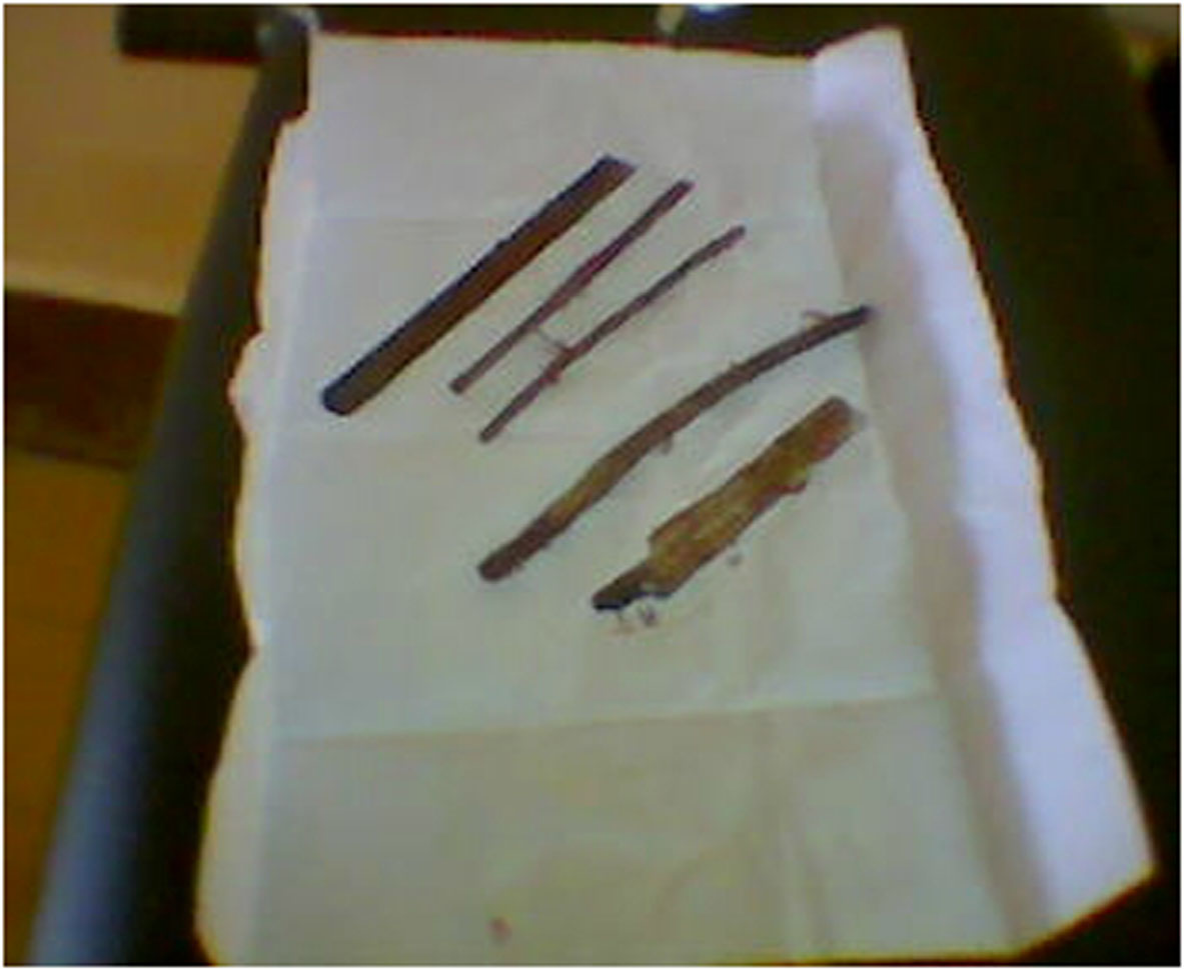
Removed foreign bodies. The picture shows two metallic and three wooden materials removed during the laparotomy

A laparotomy revealed that the materials were lodged in his sigmoid colon. There were three wooden sticks of 8–10 cm in length in addition to two sharp pieces of saw (Fig. 3). The area including the perforation in the colon was completely sealed by omentum. The materials were extracted via an enterotomy on the anti-mesenteric side of his colon and the site was closed primarily. The area was lavaged with a liter of saline and his abdomen closed. Our patient had a psychiatric consultation and was started on appropriate medications. He was discharged a week later with no major complications.

Our patient was seen on two occasions after discharge and his surgical wound and stab wound healed completely. He started regular follow-up with the psychiatric unit of our hospital and showed marked improvement.

## Discussion

Deliberate self-harm where individuals intentionally harm themselves is not uncommon in routine clinical practice but these injuries mostly do not threaten life [3]. This phenomenon typically occurs in young individuals with long-standing psychiatric problems [4]. Foreign bodies have been reported in the thorax, including the pericardium, and in various sites in the abdomen from self-inflicted wounds [5–7].

Evidence of self-mutilation and drug use have been reported in patients with severe psychiatric disorders and in incarcerated felons [8–10]. Therefore, self-mutilation should always be considered by emergency room physicians in cases of injuries in patients with unusual behavior and psychiatric disorders as well as incarcerated individuals [11, 12].

The fact that we found the foreign bodies partly lodged in the sigmoid colon of a patient who presented 3 months after the self-mutilation without having developing peritonitis is unusual. This case demonstrates that foreign bodies can enter the colon, via a self-inflicted wound as in our patient or accidentally, and the omentum can seal the area, thereby preventing the development of peritonitis.

Although foreign bodies can, as stated earlier, be found in any part of the gastrointestinal tract, they are not commonly described in the colon, as was found in our patient. Moreover, it is unlikely for any individual to inflict a wound on him or herself and introduce materials into the abdomen and not develop peritonitis, as in our patient. Our patient presented to a regular outpatient unit with no evidence of peritonitis although exploration revealed the materials to be in his sigmoid colon.

## Conclusions

This case demonstrates that foreign bodies can enter the colon, via a self-inflicted wound as in our patient or accidentally, and the omentum can seal the area, thereby preventing the development of peritonitis.
